# Non-invasive Cardiac Output Monitoring in Neonates

**DOI:** 10.3389/fped.2020.614585

**Published:** 2021-01-28

**Authors:** Roisin O'Neill, Eugene M. Dempsey, Aisling A. Garvey, Christoph E. Schwarz

**Affiliations:** ^1^Department of Neonatology, Cork University Maternity Hospital, Cork, Ireland; ^2^Department of Paediatrics and Child Health, University College Cork, Cork, Ireland; ^3^Irish Centre for Maternal and Child Health Research (INFANT) Research Centre, University College Cork, Cork, Ireland; ^4^Department of Neonatology, University Children's Hospital, Tübingen, Germany

**Keywords:** systemic blood flow, bioimpedance, electrical cardiometry, bioreactance, circulatory monitoring, transthoracic electrical biosensing technology, electrical velocimetry

## Abstract

Circulatory monitoring is currently limited to heart rate and blood pressure assessment in the majority of neonatal units globally. Non-invasive cardiac output monitoring (NiCO) in term and preterm neonates is increasing, where it has the potential to enhance our understanding and management of overall circulatory status. In this narrative review, we summarized 33 studies including almost 2,000 term and preterm neonates. The majority of studies evaluated interchangeability with echocardiography. Studies were performed in various clinical settings including the delivery room, patent ductus arteriosus assessment, patient positioning, red blood cell transfusion, and therapeutic hypothermia for hypoxic ischemic encephalopathy. This review presents an overview of NiCO in neonatal care, focusing on technical and practical aspects as well as current available evidence. We discuss potential goals for future research.

## Introduction

Monitoring and assessment of the cardiovascular system is an essential component in the care of term and preterm infants in neonatal intensive care units. Current methods of evaluation rely mainly on available bedside parameters, including blood pressure (BP), heart rate (HR), capillary refill time, and urine output, in conjunction with biochemical markers of tissue perfusion such as lactate. There are many limitations of these measurements. BP (1–4), capillary refill time (3–6), and urine output (4) do not correlate well with systemic blood flow, and HR can be easily influenced by other factors including medication, pain, and fever.

In order to accurately evaluate the cardiovascular status, two other factors need to be considered: cardiac output (CO) and systemic vascular resistance (SVR). Together, these determine systemic blood flow and subsequently end organ perfusion. The current standard measurement of CO in the neonatal unit is bedside echocardiography (Echo), as more invasive gold standard methods such as thermodilution have limited applicability and feasibility in the neonatal population. Echo use in the neonatal intensive care unit has grown exponentially over the last decade and has improved the evaluation of infants with suspected cardiovascular instability (7). However, Echo is not without limitations. Findings depict a single point in time, and extensive standardized training is required to ensure the quality of the measurements, before it can be safely implemented as a tool for cardiac assessment in neonatal intensive care units (8, 9). Despite standardization, accuracy and intra/interobserver variability remain an issue. There is also the risk of destabilizing an infant, given that a full cardiac assessment with echo can take a considerable period of time.

Given these problems, other methods need to be considered. Non-invasive cardiac output monitoring (NiCO) has the potential to provide continuous real-time measurements at the bedside. Initial studies in adults have shown it to have acceptable accuracy and precision (10, 11). However, like any new device, it should be validated. The ideal technology should fulfill certain criteria: (a) validated against gold standard; (b) accurate and precise; (c) easily applicable, non-invasive, and inexpensive; (d) continuous and easy to interpret; and (e) accurate in the presence of shunts and postnatal transition (12, 13). In reality, there is no perfect assessment method for cardiac evaluation in the neonatal population. There has been an increasing number of studies completed using NiCO in neonates over the last 10 years. The objective of this narrative review is to summarize the use of NiCO in neonatal care, focusing on both the specific areas of clinical utility and its limitations. In doing so, we aim to highlight where future research should be focused.

## Technical Background

NiCO derives from the principle of impedance. This is the measure of opposition to the flow of an alternating electrical current. The complex impedance consists of two components: the real (resistive) and the imaginary (capacitive and inductive) components, and these are known to change over time in relation to the cardiac cycle. Impedance cardiography, also known as “thoracic electrical bioimpedance,” is the study of cardiac function determined from the measurements of electrical impedance within the thorax (14). In traditional bioimpedance systems, an electrical current of known amplitude and frequency is passed through the thorax, and the change in voltages are measured. The first monitoring device was described by Kubicek et al. in 1966, who had been commissioned by NASA to create a non-invasive way of measuring cardiac output (15). Within the thorax, there are various structures and each will “impede” current differently. Blood is known to have a lower resistance to electrical current than other tissues. Impedance to electrical flow will also vary at different timepoints within the cardiac cycle, particularly during systole as blood is pumped into the aorta, causing a sharp decrease in electrical resistance within the thorax. This principle is used to estimate hemodynamic parameters such as stroke volume (SV) and CO. With advances in technology and a greater understanding of cardiac physiology, these models have been updated and adapted, and modifications made to the original mathematical algorithms to improve the quality of the results obtained. The two most recent models used for estimating CO in neonates non-invasively are electrical cardiometry (EC) and bioreactance (BR). Other technologies including signal-morphology impedance cardiology exist, but have not yet been utilized in neonates.

### Electrical Cardiometry

Bernstein and Osypka developed and described the technical background of EC, a new model for interpreting thoracic bioimpedance (16). EC uses four electrodes: two outer (head and thigh) and two inner (neck and thorax) electrodes. An alternating electrical current is applied through the two outer electrodes, and the resulting voltage is measured by the corresponding inner electrode (17). EC uses the length of the impedance vector, determined by the real and the imaginary component of impedance, and its changes in time in relation to the cardiac cycle. During diastole and prior to the aortic valve opening, red cells in the aorta are orientated in a random distribution with increased resistance to an electrical current. During systole when blood is pumped into the aorta, red cells will align resulting in a change in conductivity. By analyzing the speed of these changes, EC technology estimates peak aortic acceleration of blood flow, left ventricular (LV) pre-ejection period and ejection time (PEP and ET). This is used within the EC algorithm to derive stroke volume, and using simultaneous measurements of HR obtained from the electrocardiogram, CO is estimated. This is utilized on the Aesculon and ICON device (Osypka Medical, Berlin, Germany/Cardiotronic, San Diego, CA, USA).

### Bioreactance

BR is based on the “imaginary” component of the impedance, that is the capacitive and inductive properties of blood and biological tissue that induces phase shifts between an applied electrical current and the resulting voltage signal (10, 18, 19). This is different from bioimpedance, which uses the “resistive” component of blood and tissues to determine a change in voltage after an electrical current of known amplitude and frequency is applied across the thorax. Changes in thoracic blood volume occur with each heartbeat, and this causes an instantaneous change in the phase shift between an applied current and the measured voltage signal (19). This measured change is directly related to SV and, in conjunction with HR, can subsequently determine CO. The Cheetah NICOM/StarlingSV (Cheetah Medical Inc., Newton, MA, USA) is somewhat different from the EC technology highlighted above. Each sensor consists of two electrodes: one applying an alternating current and the other one sensing. The device measures a phase shift, also known as a time delay, between the measured thoracic voltage and the applied current (18). These phase shifts are directly related to blood flow occurring in the large thoracic arteries, with larger volumes of blood causing an increase in phase shifts. Sensors from the right side and left side of the thorax are paired together, and measurements from both sides are then averaged to estimate SV and CO.

### Important Practical Considerations for User

#### Sensor Size and Application

**Figure 1:**
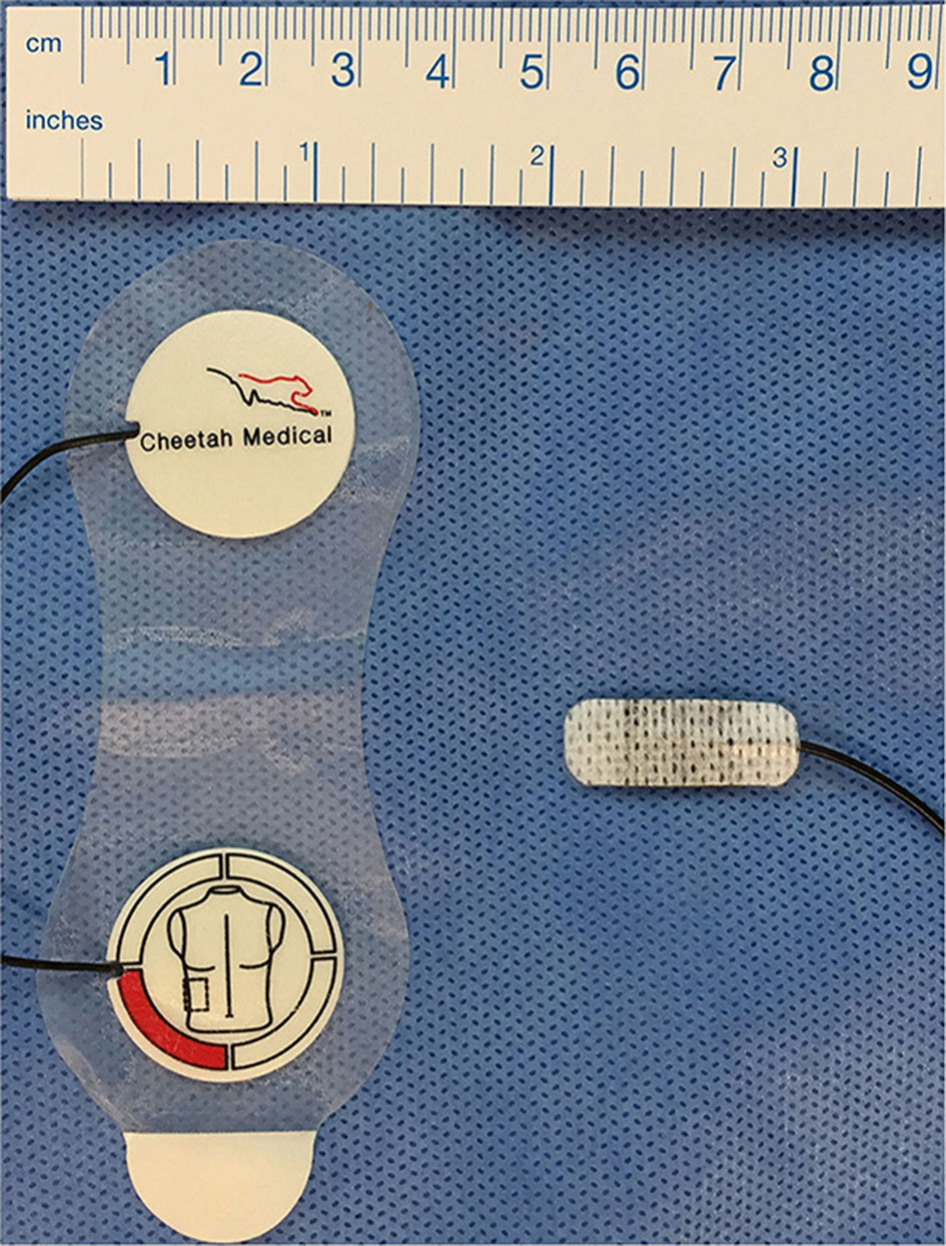
Bioreactance and electrical cardiometry electrodes. Left: Duo-electrode sensors for bioreactance monitoring (Cheetah Medical Inc., Newton, MA, USA), right: ISense neonatal sensors for electrical cardiometry monitoring (Osypka Medical, Berlin, Germany).

**Figure 2:**
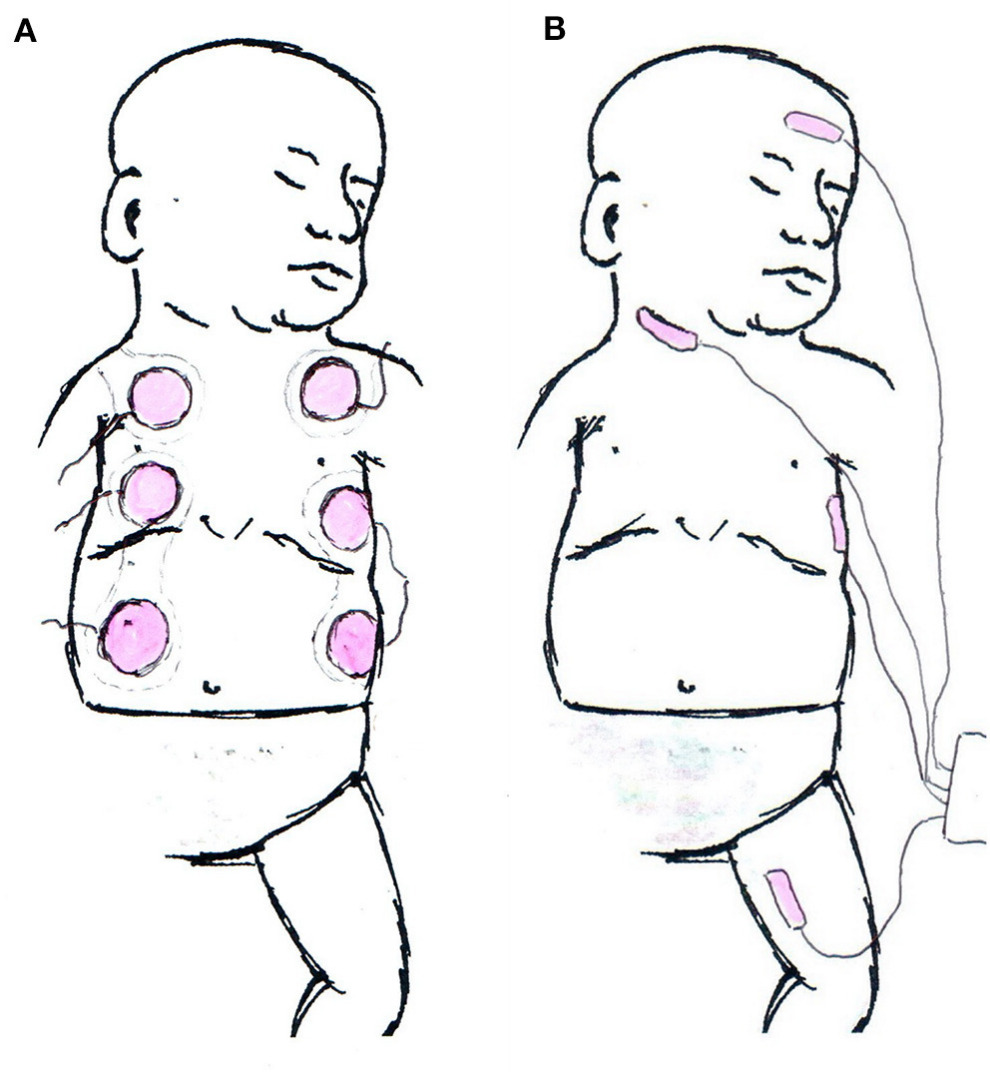
Schematic examples for electrode application. **(A)** Example of Bioreactance electrode placement in neonates. The upper part of the shoulder electrodes is crossing the shoulder and not visible from the anterior. **(B)** Current manufacturers' recommendation of sensor application in electrical cardiometry monitoring.

The sensor itself and sensor application are very different between EC and BR (Figure 1). BR uses four dual electrode stickers (~95 × 28–40 mm each with two circular shaped electrodes with a diameter of 25 mm), positioned in a “box” surrounding the heart: two sensors on the right side and two on the left side of the body (Figure 2A). Given the relatively large surface area of these sensors, application in newborns is challenging. Different solutions have been published including cutting down the adhesive component of the sensors and altering the placement positions (20–23). On the other hand, EC has specific neonatal sensors, which are smaller in size (25 × 10 mm). Previously, sensors have been placed on the head, left side of neck, left thorax (at the level of the xyphoid), and left outer thigh. Recently, there have been adaptions made by the manufacturer, with regard to the ideal placement of sensors. They now recommend that the neck probe should be placed on the right side of the neck, and that the leg-probe should be placed on the inner thigh for improved accuracy (Figure 2B).

#### Calibration/Adaption for Bodyweight and Length

EC uses an internal calibration, which takes into account the neonate's bodyweight and length for SV and CO estimates, to adjust for effects on distance between sensors (17). This is necessary to address different distances between emitters and sensors regarding the size of the infants. BR does not take into account differences in body length directly, but the algorithm accounts for age, gender, and body size based on data from adult population (18).

#### Signal Quality

The StarlingSV/Cheetah does not display or log values during periods with low signal quality. Rather, no value is displayed. EC devices display signal quality estimated automatically as a percentage representing the proportion of good signal quality cardiac cycles obtained over the preceding 10 cardiac cycles.

#### Frequency of Data Logging

Unaveraged data with beat-to-beat resolution is not available on the StarlingSV at present. The minimum logging interval is now 4 s. Internally, the StarlingSV is estimating the CO every beat, but data is averaged over the preceding 24 s. This averaging interval is divided into 8 s episodes, and within each episode, a minimum of two analyzable beats are required (direct communication with the distributor). However, all neonatal studies using BR in this review, used data logged and subsequently averaged over a minute by minute basis. In contrast, EC devices provide beat-to-beat values, as well as various averaging and logging intervals starting with a minimum of 5 s. The principle of unaveraged beat-to-beat resolution may be more relevant in the research setting, rather than in day to day clinical care.

## Feasibility Studies

These studies were primarily focused on the use of NiCO in the delivery room. They investigated whether it was possible to both apply the sensors, and to obtain values in the immediate postnatal period. Katheria et al. (24) looked at the feasibility of using EC within the first 5 min after delivery in 20 vaginally delivered term infants with an intact cord. The first signal/reading was available at a median time of 89 s of life [IQR [83, 116]]. No data was recorded in the first minute, and ~50% of the subjects had data available between 1 and 2 min of life. As mentioned previously, the EC algorithm relies on both weight and length to calibrate accurately the absolute values of CO. Given this was not available immediately after birth, the measurements recorded are only useful as a trend monitor. Freidl et al. (25) also used EC to monitor term infants in the delivery room for the first 15 min of life. They determined questionable feasibility, given that 76.2% of the measurements had to be excluded because of a signal quality index <80%. More recently, a study carried out by our hospital group evaluated the role of BR in the delivery room on a cohort of 49 babies. Values were obtained at a median time of 3 min from application of sensors. Again, trend values were obtained over the first 15–20 min of life and compared to values obtained at ~2 h of age (26).

## Accuracy Studies

### Interchangeability to Echocardiography

The vast majority of publications address the accuracy of EC or BR, as the direct interchangeability to Echo (as the reference method). A range of prospective observational studies have been carried out, and collectively include over 300 preterm and more than 100 term infants (Table 1). However, it is important to understand the implications of comparing EC/BR to a reference method such as Echo, which also has shortcomings in both precision and accuracy. The limit of precision of Echo compared with the gold standard method of dilution technique is ~30%, which is within the clinically accepted range (38). This is also in line with comparison of Echo to cardiac magnetic resonance imaging studies revealing a repeatability index of 28.2% (39).

**Table 1:** Accuracy studies.

**Study**	**Technology**	**Population**	**Age at measurement [days]**	**Interchangeability/Systematic bias**
Noori et al. (17)	EC	20 “healthy” term	<3	Not interchangeable, no systematic bias
Grollmuss et al. (27)	EC	24 newborns with transposition of the great arteries after switch-OP	10 (3–29)	Interchangeable, systematic over-reading
Grollmuss et al. (28)	EC	28 preterm	15 (1–48)	Interchangeable, systematic over-reading
Song et al. (29)	EC	40 preterm	<1.5	Not interchangeable except infants in room air, systematic under-reading except for over-reading during HFV
Blohm et al. (30)	EC	72 total aged 2 days−17 years (subgroup 26 preterm)	Preterm subgroup 14.6 (3.7–40.2)	Not interchangeable in preterm infants subgroup, systematic under-reading
Boet et al. (31)	EC	59 neonates	NA	NR
Torigoe et al. (32)	EC	28 preterm	4 (1–13)	Interchangeable, no systematic bias
Boet et al. (33)	EC	79 preterm	NA	NR Trend to overestimate
Boet et al. (34)	EC	30 NICU/PICU patients during transportation	29 (41)	“Comparable reliability,” systematic over-reading
Hsu et al. (35)	EC	36 preterm with hsPDA	6 (2–22)	Interchangeable, but decreased for respiratory support with HFV, no systematic bias
Weisz et al. (21)	BR	10 infants with GA >31 weeks	NA	Interchangeable, systematic under-reading
Weisz et al. (36)	BR	25 preterm post PDA ligation	~5–6 weeks	Interchangeable, systematic under-reading
Forman et al. (37)	BR	8 term infants undergoing TH for HIE	<5	%Error NR, systematic under-reading
Van Wyk et al. (20)	BR	63 preterm	<3	Not interchangeable, systematic under-reading

*EC, Electrical Cardiometry; BR, BioReactance; GA, Gestational Age; LV, left ventricular; CO, cardiac output; HFV, High Frequency Ventilation Age in measurements presented in days as mean (SD), median (IQR) or < threshold as reported. Criterion for interchangeability is combined percentage error <42%; NR is stated if percentage error was not reported*.

Critchley et al. (40) published a meta-analysis of studies which had used bias and precision to compare CO estimation techniques (NiCO vs. Echo). The authors identified a 30% percentage error to be clinically acceptable (40). In a systematic review and meta-analysis of CO measurements in children, the NiCO method was identified as being the most accurate and precise, when compared to other non-invasive or invasive techniques used to monitor CO (41). However, a subgroup analysis looking at age below 1 year identified a pooled bias of −0.08 L/min and a percentage error of 33.2% compared to Echo (41). This would suggest non-interchangeability in this age group. Of note, this analysis was limited to only two studies in term and near-term infants (17, 21). A more recent systematic review focusing on EC (42) included neonates in a subgroup of pediatric studies (2 studies out of 11) (17, 32, 42). Van Wyk et al. (20) recently summarized the available literature on reproducibility of NiCO and Echo addressing the limited precision of Echo. In line with the previous definition, interchangeability of the two methods was assumed when the combined percentage error is <42% (equal to √[30^2^ + 30^2^]). As part of this review, we identified nine studies on EC (17, 27–33, 35) and four studies on BR (21, 36, 37) in neonatal care. In line with Van Wyk et al. (20) we conclude that the NiCO is not interchangeable with Echo for CO measurement. Interpreting NiCO derived values with thresholds based on Echo values is problematic. As EC monitors display and log estimates of signal quality, studies investigating interchangeability with Echo used cutoffs for signal quality index of >70%, thus accounting for poor signal quality (17, 32, 33, 35). This might explain differences in reproducibility in EC compared to BR studies.

### Confounding Factors on Accuracy

Respiratory support in preterm infants is common and often includes both non-invasive and invasive ventilation techniques. These have been shown to affect the accuracy of EC and BR, especially during periods of high-frequency oscillation (20, 21, 29). Other co-morbidities related to immaturity, including shunts such as a PDA, have also been identified as confounders (32). Depending on the degree and the direction of shunting, this can either lead to an underestimate (right-to-left) or an overestimate (left-to-right) of effective systemic blood flow, particularly when using left ventricular CO as a surrogate marker for this (43). In addition, the more common left-to-right shunt through a patent foramen ovale results in an overestimation of systemic venous return by increasing right ventricular (RV) CO (36, 44, 45). In a comparison study of EC with Echo in term infants, there was no significant difference in the bias or precision between the two methods, in patients with or without a hemodynamically significant PDA (defined as a diameter > 2 mm) (17). On the other hand, in a group of preterm infants using BR, the presence of a PDA was found to significantly affect the accuracy of CO (20). In the same study, they also found that the level of CO value itself influenced the accuracy of the measurement. Low and high CO situations were found to be less accurate compared to “normal” output ranges (20). As these low or high output states are clinically relevant and important to identify and treat, this may potentially limit the diagnostic value of NiCO.

## Investigated Clinical Conditions/Situations

In addition to those accuracy and validation studies discussed, EC and BR reported that neonatal studies have also included ~1,000 preterm and 400 late preterm/term infants across a range of clinical settings. In Table 2, studies investigating NiCO-derived estimates for SV and/or CO are summarized.

**Table 2:** NiCO in various scenarios.

**Topic**	**Study**	**Technology**	***N***	**Population**	**Primary objective**	**Finding**
**Delivery room and postnatal management**						
	Katheria et al. (46)	EC	140	Preterm	RCT comparing Delayed Cord Clamping vs. Umbilical Cord Milking	No differences in CO measured by EC between two groups
	Katheria et al. (24)	EC	20	Term	Feasibility of EC in Delivery room	Feasible, but challenging
	Katheria et al. (47)	EC	125	Preterm	RCT comparing ventilation during Delayed Cord Clamping vs. Delayed Cord Clamping only and effects on hematocrit in the first 24 h	No differences in SV or CO measured by EC between groups
	Freidl et al. (25)	EC	100	Term	Early transition within 15 min after birth	Feasible, but challenging
	McCarthy et al. (26)	BR	49	Term	Early transition within 15 min after birth and at 2 h of life	Feasible, but challenging
**Transition**						
	Cappelleri et al. (48)	BR	45	Preterm	Myocardial function during the first 48 h	CO and SV found to increase over the first 48 h of life
	Miletin et al. (23)	BR	39	Preterm	CO within the first 48 h in relation to adverse outcome	Adverse Outcome is associated with low CO in the first hours followed by high CO in the second 24 h
	Hsu et al. (49)	EC	280	Term and Preterm	Normative data > 72 h for EC derived CO, SV (as well as TFC, ICON, and SVR)	Description of EC values beyond 72 h in various age groups
**Patent ductus arteriosus**						
	Lien et al. (50)	EC	30	Preterm undergoing PDA ligation	Hemodynamic changes before, during, and after PDA ligation	Significant decrease in SV and CO immediately following ligation, compared to pre-surgery baseline
	Katheria et al. (51)	EC	292	Preterm 55 treated for PDA	Prediction of PDA closure with 24 h of age	EC derived CO in the first day is not predictive for hsPDA
	Rodríguez Sánchez de la Blanca et al. (52)	EC	18	Preterm with hsPDA	Hemodynamic changes before, during, and after treatment	Significant decrease in CO 72 h after treatment
	Hsu et al. (53)	EC	18	Preterm PDA, Ibuprofen non- (9) or responders (9)	Hemodynamic effects of Ibuprofen for PDA	Non-responders had higher CO compared to responders
**Effects of positioning**						
	Ma et al. (54)	EC	30	Preterm and Term	Cardiovascular response to Positioning	Decrease SV/CO in prone position compared to supine
	Wu et al. (55)	EC	34	Term	Cardiovascular response to Positioning	Decrease SV/CO in prone position compared to supine
	Paviotti et al. (56)	EC	32	Term and preterm	Cardiovascular response to Positioning	Decrease SV/CO in supine position compared to left-lateral position
**Hemodynamic monitoring during transport**						
	Boet et al. (34)	EC	30	Preterm and Term	Inter-center transfer	SV monitoring during transport is feasible and reliable
**Hemodynamic effects of medication**						
	Katheria et al. (57)	EC	21	Preterm	Early vs. late Caffeine	No differences in CO between groups from 2 to 24 h of age
	Katheria et al. (58)	EC	36	Preterm	Hemodynamic effects of Sodium Bicarbonate	No differences found in CO up to 80 min following administration
	Truong et al. (59)	EC	35	NICU patients	Hemodynamic effects of premedication for neonatal intubation	No differences in CO before and after premedication for intubation
**Effects of anemia/transfusion**						
	Weaver et al. (60)	EC	75	Preterm anemic (35) vs. no-anemic (40)	Hemodynamic effects of RBC transfusion	Increase in CO between 0 and 120 min post transfusion.
	Jain et al. (61)	EC	30	Preterm anemia	Hemodynamic effects of RBC transfusion	No difference in CO measurements in hour pre and post transfusion
**Effects of HIE, TH, and rewarming**						
	Wu et al. (62)	EC	20	Term undergoing TH for HIE	Hemodynamic effects of rewarming	CO found to increase during rewarming
	Eriksen et al. (63)	EC	25	Term 15 asphyxiated vs. 10 controls	Hemodynamic effects of early (1st 6 h) of TH and to assess the effect of low CO on lactate clearance	CO found to be reduced during TH
	Forman et al. (37)	BR	20	Term undergoing TH for HIE	Feasibility and reliability of multimodal non-invasive monitoring during TH and rewarming for HIE	CO found to increase during rewarming

*EC, Electrical Cardiometry; BR, BioReactance; GA, Gestational Age; CO, cardiac output; SV, Stroke volume; TFC, Thoracic Fluid Content; ICON, Index of CONtractility; NICU, Neonatal Intensive Care Unit; PICU, Pediatric Intensive Care Unit; PDA, Patent Ductus Arteriosus; SVR, Systemic Vascular Resistance; TH, Therapeutic Hypothermia; HIE, Hypoxic Ischemic Encephalopathy; RCT, Randomized Controlled Trial; RBC, Red Blood Cell*.

### Delivery Room Management

Katheria et al. (24) used EC to measure CO within the first 5 min after birth in term infants. Freidl et al. (25) extended this to the first 15 min after birth. As we have previously highlighted, BR has also been used in the delivery room (26). While there was no direct comparison to Echo in any of these studies, the reported results are comparable to previous data using Echo in the delivery room (64). Delivery room EC is also limited by the fact that the algorithms require birth weight and body length to determine absolute values, as well as body weight indexed values, and thus is limited to trend changes over this time period.

Cord management strategies are known to impact circulatory parameters in later postnatal transition. In randomized controlled trials of preterm infants <32 weeks gestational age (GA) receiving umbilical cord milking (UCM) vs. immediate cord clamping, infants randomized to UCM had higher superior vena cava flow (SVC-flow) and RV CO measured using Echo, but no differences were found for NiCO-derived parameters within the first day of life (46, 65).

A separate study looking at two groups of preterm infants randomized to either respiratory support during delayed cord clamping (DCC) (60 s) or DCC without respiratory support, showed no difference in EC-derived hemodynamic measurements in the first day of life (47).

### Transition

Hemodynamic monitoring in the first few days of life is particularly challenging given the presence of persistent shunts, and the complex physiological changes that occur in the transition from fetal to neonatal circulation (7). Cappelleri et al. (48) investigated changes in left ventricular output in mid-to late preterm infants within the first 2 days of life. Using BR to estimate LV CO and SV, they reported an incremental increase in both these parameters, but with a stable HR throughout (48). In a recent study Miletin et al. (23) published BR-derived values for more immature preterm infants between 6 and 48 h of life in relation to clinically relevant outcomes. In line with Cappelleri et al. (48) they reported lower CO at 6 h, which increased over the second day of life. Interestingly, this increase was found to be associated with an increased incidence of intraventricular hemorrhage suggesting reperfusion injury as a pathophysiological explanation. Beyond early transition, Hsu et al. (49) published reference values in term and preterm infants without PDA >72 h postnatally. Whether NiCO alone (ClinicalTrials.gov NCT04064177), or integrated into multimodal monitoring (ClinicalTrial.gov NCT04538079), results in improved clinical outcome in preterm infants is currently under evaluation.

### Patent Ductus Arteriosus

PDA is still one of the most controversial areas of preterm management. EC was used to evaluate the early (<24 h) prediction of significant PDA in preterm infants (51). This so-called hemodynamically significant (hs) PDA is not clearly defined. However, the authors found that Echo-derived LV CO was predictive for hsPDA. Infants treated for a PDA were found to have lower mean BP, CO, and SV compared to untreated, but after adjustment for GA and birth weight, all parameters except mean BP were found to be non-significant. In contrast, Rodríguez et al. (52) monitored infants undergoing treatment of PDA (three ibuprofen doses) using EC. They reported a significant decrease in CO indexed for body weight (0.24 vs. 0.29 L/kg/min; P 0.03) after 72 h. Hsu et al. (53) found infants with PDA had higher pre-treatment baseline CO determined by EC compared to non- PDA infants. Interestingly, non-responders to medical treatment with ibuprofen had higher CO compared to responders. EC has been utilized peri-operatively in PDA ligation and may improve management of post ligation syndrome (50).

### Positioning

Three small cohort studies comparing the effect of positioning of the infant on CO measurements revealed differences in prone or left-lateral and supine position (54–56). Ma et al. (54) found decreased SV and CO in prone position compared to supine position using EC. This was confirmed by Wu et al. (55) using both EV and Echo. These results may have important implications for clinical use, particularly as many preterm infants are often nursed prone. Paviotti et al. (56) compared left lateral position to the supine position using EC technology. Both SV and CO were found to be decreased significantly in the supine vs. the left-lateral position.

### Hemodynamic Monitoring During Transport

With ongoing centralization of neonatal care, the transportation of unstable infants born in level one or level two centers will increase. Therefore, hemodynamic monitoring during transport may facilitate more appropriate management in this specific situation. During inter-center transfer, a method of reliable monitoring of circulatory status might have an important impact on transport management decisions. SV monitoring with EC was found to be feasible in 30 infants during inter-center transfer (34). EC-derived SV was higher compared to Echo, but both methods were identified as reliable. Whether continuous NiCO results in improved post transport outcome has yet to be determined and is another area that warrants further study.

### Studies Evaluating Hemodynamic Effects of Medications

Three studies have used EC for hemodynamic monitoring in neonates receiving medications including caffeine (57), sodium bicarbonate (58), and premedication for intubation (59).

In 2015, Katheria et al. used EC in a small group (*n* = 21) of non-intubated preterm infants, who were either randomized to early (<2 h) or late (12 h) administration of caffeine after birth. Numerous indicators of systemic blood flow including SVC flow, LV, and RV CO were estimated by Echo at a mean time of 6 h. Serial measurements including SV, CO, mean BP, and HR were also recorded between 2 and 24 h of life using both EC and an umbilical artery catheter (57). No significant differences were found between the two groups in both LV CO as measured by Echo or CO, which was recorded with EC. Only a small number of studies have looked at the cardiovascular effects of caffeine in neonates. Some studies suggest an improvement in CO (66, 67), while others have suggested no significant change (68, 69). Given the timing of this study, it is possible that Echo and EC-derived CO measurements may have been impacted by large left to right ductal shunts, which are often a feature of the early neonatal transitional circulation.

Katheria also looked at 36 preterm infants (mean GA 26.3 weeks) who received NaHO_3_ in the first 24 h of life for metabolic acidosis. They recorded average HR, BP, CO, and cerebral oxygen tissue saturation (CrSO_2_), over 10-min intervals up until 80 min post NaHO_3_ administration (58). No comparative measurements of CO were measured using Echo over the period of observation. One study including 16 neonates demonstrated that sodium bicarbonate induced a significant but transient rise in CO, aortic blood flow velocity, and systolic BP (70). Other studies in adult populations have shown little cardiovascular benefits (71, 72).

More recently, Truong used EC to assess the hemodynamic effects of premedication in 37 infants (mean GA 31.6) requiring intubation. A combination of atropine, followed by fentanyl/morphine and finally cisatracurium was used in 36/37 infants. There was no significant difference found in CO before and after premedication. However, 17 infants did have a ≥20% drop in CO after intubation. Ten infants also had a ≥20% drop in mean BP. This decline in BP did not correlate with any fall off in CO. The use of Echo to assess cardiovascular changes during premedication and intubation is not feasible. NiCO offers an alternative non-invasive method for assessing hemodynamic changes prior to and during intubation.

### Hemodynamic Effects of Red Blood Cell Transfusion

There have been two studies using EC in preterm neonates receiving red blood cell transfusions (RBC) (60, 61). Weaver et al. (60) looked at the hemodynamic characteristics of 75 preterm infants, and compared those who had anemia requiring transfusion (*n* = 35), with a control group who were not anemic (*n* = 40). Only stable infants were included in the analysis, and any infants on mechanical ventilation, or those with suspected altered perfusion were excluded. Measurements including CO, SV, HR variability (HRV), and complexity (HRC) were recorded using the ICON monitor and were continued for a minimum of 4 h in both groups of patients. The mean adjusted GA was similar in both groups, 32–33 weeks. There was a statistically significant difference in CO between the non-transfused group and the transfused group prior to treatment, 0.28 vs. 0.17 L/min, respectively. SV was also higher in the non-transfused group but did not reach statistical significance. For the group that was transfused, they found that CO increased over time and that this was statistically significant at all time points between 0 and 120 min. Despite this increase, CO measurements in the transfused group still remained consistently lower than the non-transfused group.

A second study by Jain et al. (61) measured CO and near-infrared spectroscopy (NIRS)-derived CrSO_2_ in 27 preterm infants in the hour prior to and the hour post transfusion. Pre-transfusion oxygen delivery index (ODI) was also calculated using the formula: Hemoglobin (Hb) [g/dl] × CO [L/kg/min]. There was no statistical difference identified in CO before and after transfusion. In addition, the pre-transfusion CO and Hb did not correlate with CrSO_2_ or in the change in CrSO_2_ following transfusion. It was the pre-transfusion ODI that was found to be a greater determinant of tissue perfusion. A significant correlation was found between ODI and both pre-transfusion CrSO_2_, and the change in CrSO_2_ after transfusion. This would suggest a role of CO monitoring to identify those preterm infants who are most likely to benefit from RBC transfusion. Numerous studies have reported a decrease in CO measured by echo following transfusion (73–75). Saleemi et al. (76) found no significant changes in load-dependent parameters, but instead found an improvement in myocardial contractility following transfusions. As cardiac monitoring was only continued for a short time after transfusion in the study by Weaver, the increase found in CO and HRV in the transfused group may not fully reflect the longer-term effects of RBC transfusions on the cardiovascular system. Further studies with NiCO in the post-transfusion period would be beneficial.

### Hypoxic Ischemic Encephalopathy, Therapeutic Hypothermia, and Rewarming

Outcome in infants with hypoxic ischemic encephalopathy (HIE) has improved significantly since the introduction in 2008 of therapeutic hypothermia (TH) as standard of care for infants with moderate and severe grades of encephalopathy (77). Despite this, a significant number of infants continue to have poor neurodevelopmental outcome at follow-up (77–79). Adjunct therapies are currently under investigation (80). HIE has been shown to result in myocardial ischemia and have a transient effect of myocardial function, which may further complicate cerebral perfusion (81–85). Although BP monitoring is widely available, changes in CO may occur independent of changes in BP (37, 86). NiCO monitoring has the potential to provide a continuous, non-invasive measurement of CO in these infants. To date, three studies have assessed the use of NiCO in infants with HIE, specifically looking at the effect of TH on cardiac function (37, 62, 63).

Eriksen et al. (63) used EC to examine CO for the first 6 h of life in 15 infants undergoing TH for moderate and severe grades of HIE compared with 10 healthy term controls. NiCO was also used to assess the effect of low CO on lactate clearance during the same timeframe. Five infants with HIE had NiCO measurements available prior to initiation of TH, which showed an impairment in CO and SV when compared to healthy term controls. CO was reduced in all infants during TH compared with controls, but this was mainly due to a reduction in HR. In infants with HIE, rate of clearance of lactate did not correlate with CO. Of note, the rate of lactate clearance correlated with the highest Thompson score. Forman et al. (37) used BR to assess the effects of TH on CO. They recruited 20 infants undergoing TH for moderate and severe grades of encephalopathy and recorded NIRS and NiCO measurements during TH and the rewarming period. Eight infants also had serial point of care Echos performed during the monitoring period. CO increased during the rewarming period, and this was predominantly due to an increase in heart rate. There was a strong correlation between BR and Echo-derived measurements of CO; however, NiCO measurements of CO were consistently 27% lower than Echo measurements, similar to previous comparison studies (21, 36).

Wu et al. (62) used EC and Echo to examine changes in CO during the rewarming period in 20 infants with moderate and severe grades of HIE. Both EC and Echo derived measurements of CO increased during the rewarming period from 153 ± 43 ml/kg/min to 197 ± 42 ml/kg/min and 149 ± 35 ml/kg/min to 179 ± 34 ml/kg/min, respectively. HR increased significantly, and SV remained unchanged. Both systemic vascular resistance and mean arterial blood pressure decreased during the rewarming period but did not meet GA thresholds for intervention.

TH has a significant effect on HR and thus CO. Whether this is a protective mechanism or a response to a decrease in the basal metabolic rate requires further research. NiCO monitoring during TH is feasible. NiCO measures correlate with previously described Echo measures of CO and reflect expected hemodynamic changes during TH (86, 87).

### Studies Evaluating Other Parameters Derived by NiCO Monitors

EC devices provide a variety of other circulatory parameters, such as Cardiac contractility estimated as an Index of Contractility (“ICON”–value), its Variation of Index (VIC), LV Systolic Time Ratio (LV-STR = LVPEP/LVET), Thoracic Fluid Content (TFC), Stroke Volume Variation (SVV). The use of EC-derived TFC in respiratory distress syndrome diagnosis and management (88) requires further investigation. The same is required for the use of HRV in diagnosing sepsis or infection (89). In line with EC, BR devices display estimates of change in SV index, TFC, and LVET. Integrated in the NiCO devices, these parameters are available at the bedside in real-time potentially improving diagnosis and subsequent treatment. However, studies with larger sample sizes are needed before introduction into routine clinical care.

## Discussion

Neonates, and in particular preterm infants, have a very unique and complex cardiovascular system in the first days of life, which is inherently different to adult or pediatric populations. Monitoring is also very different, as the most accurate and precise methods published, such as thermodilution and cardiac MRI are just not feasible in this age group. Transthoracic Echo is the current reference for measuring cardiac performance in the neonatal population, but it has various limitations. In addition, there are conflicting reports with regard to the accuracy of Echo itself. A systematic review by Wetterslev et al. (90) comparing echocardiography to thermodilution in mainly adult studies, suggested that the two techniques are not interchangeable. Of note, many of those studies included transesophageal Echo, which is not routinely used in neonates (90). A follow-up systematic review and meta-analysis by Zhang et al. (91) concluded that there was no significant difference between the two methods. However, they also found that in certain situations, such as high CO or physiological structural changes, the accuracy of CO by echocardiography was questionable. This is obviously very relevant to the neonatal population who are subject to both these issues.

The studies to date using EC/BR in the neonatal population have been carried out only in a research setting, and many have focused on the interchangeability with Echo. However, given the limitations of Echo, comparing these two methods and calculating the bias is overly simplistic. Critchley et al. (40) eluded to this point and outlined the need to present percentage errors and limits of agreement to fully evaluate any new techniques in cardiac monitoring. They proposed that when looking at any new methods for cardiac evaluation, an acceptable limit of agreement (LOA) would be ±30%. However, these acceptable LOA are also reliant on the reference method having an acceptable accuracy of ±10–20%, which is not associated with Echo (20, 40).

We have outlined a range of studies including over 2,000 mostly preterm infants where EC and BR have been used. Feasibility remains a concern and many studies have reported problems with the size of the adhesive sensors particularly those using bioreactance (20, 21, 36, 37). The use of EC/BR in the delivery room has also been problematic with both time delays in recording and signal quality issues (24, 25). The use of these adhesives over a more extended time period has yet to be determined.

The accuracy of EC and BR is very difficult to determine without comparison to the more well-accepted methods such as thermodilution/pulmonary artery catheterization. However, this is neither safe nor ethically acceptable in this vulnerable group. Studies in neonatal animal models may allow us to better assess the precision and accuracy of these models. Other factors such as PDAs, respiratory support, and level of cardiac output have all been shown to significantly affect bias (20). The effects of other confounders on accuracy and precision would need to be explored fully in further studies. This may also provide identification of physiological vs. pathological values, rather than just a direct comparison with values obtained by other methods for cardiac output monitoring.

Non-invasive methods for cardiac evaluation in neonates are probably more important than in other population groups, given the limited tools we have in practice at present. Studies to date using EC/BR suggest that they cannot substitute Echo, but that they may offer some benefits in trend monitoring. The focus of many new cardiac monitoring techniques is comparison to the “gold standard.” Feldman explained the need to move beyond this approach and instead focus on whether these new technologies can improve clinical decision making and ultimately patient outcome (92). This idea was followed by Biasis et al. (93) who pointed out that variations in cardiac output are probably more beneficial than an absolute value in most cases. They also found that positive patient outcomes using less accurate hemodynamic monitoring systems were often associated with devices that used specific therapeutic protocols. They concluded that accuracy is important and necessary, but that they also must be accompanied by outcome studies. The optimal technique—yet to be identified—should be evaluated stepwise: starting with its accuracy compared to “gold standard” and including its confounders, relation to clinically relevant outcome, estimate values for decision making (including sensitivity and specificity of cut-offs and its confounders), and last but not least inclusion in therapeutic protocols and its effects on the patient's outcomes (93).

## Conclusion

Despite questionable interchangeability with Echo and evidence for its various confounders, both EC and BR are frequently used in various research settings. NiCO technology provides non-invasive continuous hemodynamic monitoring. As a result, this technology has the potential to positively impact on circulatory monitoring, management, and ultimately patient outcome. However, normative data or intervention thresholds from echocardiography should not be used in the interpretation of NiCO-derived SV and CO. Its implementation in treatment algorithms and its effect on clinically relevant short- and long-term outcomes need to be addressed in future research. Until this evidence is available, it should not be used in routine neonatal clinical practice.

## Author Contributions

CES and EMD conceived and designed the review. CES, RO'N, AAG, and EMD contributed to the drafting of the initial and the revised manuscript, critically revised the manuscript for important intellectual content, agreed on the final manuscript, and approved its submission for publication. All authors contributed to the article and approved the submitted version.

## Conflict of Interest

CES and EMD received an ICON device (Osypka Medical, Berlin, Germany) free of charge for 2 years. The remaining authors declare that the research was conducted in the absence of any commercial or financial relationships that could be construed as a potential conflict of interest.
